# A randomised controlled trial comparing palate surgery at 6 months versus 12 months of age (the TOPS trial): a statistical analysis plan

**DOI:** 10.1186/s13063-020-04886-y

**Published:** 2021-01-04

**Authors:** Elizabeth J. Conroy, Rachael Cooper, William Shaw, Christina Persson, Elisabeth Willadsen, Kevin J. Munro, Paula R. Williamson, Gunvor Semb, Tanya Walsh, Carrol Gamble, On behalf of the TOPS trial management group

**Affiliations:** 1 Liverpool Clinical Trials Centre, University of Liverpool, a member of Liverpool Health Partners, Institute of Child Health, Alder Hey Children’s NHS Foundation Trust, Liverpool, L12 2AP UK; 2 School of Medical Sciences, Division of Dentistry, The University of Manchester, Manchester, UK; 3 Institute of Neuroscience and Physiology, Speech and Language Pathology Unit, Sahlgrenska Academy, University of Gothenburg, Gothenburg, Sweden; 4 Department of Nordic Studies and Linguistics, University of Copenhagen, Copenhagen, Denmark; 5 Manchester Centre for Audiology and Deafness, School of Health Sciences, The University of Manchester, Manchester, UK; 6 Manchester University Hospitals NHS Foundation Trust, Manchester Academic Health Science Centre, Manchester, UK

**Keywords:** Randomised controlled trial, Complex intervention, Surgical intervention, Trial design, Trial analysis, Statistical analysis plan, Palatal surgery, Unilateral cleft palate, Sommerlad technique, Velopharyngeal function

## Abstract

**Background:**

Cleft palate is among the most common birth abnormalities. The success of primary surgery in the early months of life is crucial for successful feeding, hearing, dental development, and facial growth. Over recent decades, age at palatal surgery in infancy has reduced. The Timing Of Primary Surgery for cleft palate (TOPS) trial aims to determine whether, in infants with cleft palate, it is better to perform primary surgery at age 6 or 12 months (corrected for gestational age).

**Methods/design:**

The TOPS trial is an international, two-arm, parallel group, randomised controlled trial. The primary outcome is insufficient velopharyngeal function at 5 years of age. Secondary outcomes, measured at 12 months, 3 years, and 5 years of age, include measures of speech development, safety of the procedure, hearing level, middle ear function, dentofacial development, and growth. The analysis approaches for primary and secondary outcomes are described here, as are the descriptive statistics which will be reported. The TOPS protocol has been published previously.

**Discussion:**

This paper provides details of the planned statistical analyses for the TOPS trial and will reduce the risk of outcome reporting bias and data-driven results.

**Trial registration:**

ClinicalTrials.gov NCT00993551. Registered on 9 October 2009.

**Supplementary Information:**

The online version contains supplementary material available at 10.1186/s13063-020-04886-y.

## Background

Clefts of the lip and/or palate are among the most common birth anomalies, occurring with an incidence of 1 in 600 births [1]. The timing of palatal surgery has been a controversial issue since the 1930s [2]. Traditionally, rationale for delaying hard palate surgery was partly based on the belief that postponing the trauma of palatal closure may reduce maxillary growth disturbance. However, there is little evidence that facial skeletal growth in individuals with isolated cleft palate is substantially affected by different surgical protocols, though maxillary arch form, especially transversely, may be affected [3–6].

Over recent decades, the age at which palatal surgery is carried out has reduced. This has led to one-stage palatal closure within 12 months of age at cleft units in Europe and the USA. Protagonists of early closure of the palatal cleft have proposed that since speech is a learnt behaviour, the sooner an intact anatomy is created, the better [7–10]. As yet, however, there is no evidence that early surgery would lead to better speech development.

The Timing of Primary Surgery for cleft palate (TOPS) trial is an international, two-arm, parallel group, randomised controlled trial designed to determine whether, in infants with isolated cleft palate, it is better to perform primary surgery at age 6 or 12 months (corrected for gestational age). This research will investigate the effect of the timing of surgery by assessing and comparing speech development outcomes measured across 12 months, 3 years, and 5 years of age. In addition, secondary outcomes include safety of the procedure, hearing level, middle ear function, dentofacial development, and growth. The protocol paper for the TOPS trial has been published previously [1]; the aim of this paper is to report in detail the statistical analysis plan. This paper has been prepared according to the published guidelines on the content of statistical analysis plans [11].

## Methods and design

### Trial design

TOPS is an international, multi-centre trial using a parallel arm design aiming to detect whether surgery at 6 months is superior to surgery at 12 months. Infants with a diagnosis of cleft palate are randomised to receive primary surgery for cleft palate using a standardised technique (the Sommerlad technique [12]) at either 6 months or 12 months (corrected for gestational age). Eligible patients are randomised on a 1:1 basis using minimisation routine, incorporating a random element to reduce predictability, to balance the two groups by surgeon (*n* = 24) and size of cleft (soft palate only vs. soft and hard palate). The nature of the intervention prevented this trial from being blind to participants or their carers. However, speech outcomes, at ages 12 months, 3 years, and 5 years, will be rated blind to the randomly allocated group by independent assessment of speech recordings taken at visit. The primary outcome is assessed at age 5 years with secondary outcomes assessed 48 h and 30 days post-surgery and at age 12 months, 3 years, and 5 years. Full details of the trial design, study population, and study procedures have been published previously [1].

The trial is registered with the ClinicalTrials.gov Identifier: NCT00993551 (registered: 9 October 2009).

### Objectives

The primary objective is to determine whether surgery for cleft palate, using the Sommerlad technique, at age 6 months when compared to surgery at age 12 months improves velopharyngeal function at age 5 years. Secondary research objectives include whether timing of surgery improves speech development, safety of the procedure, hearing level, middle ear function, dentofacial development, and growth.

### Outcomes

#### Primary outcome

The primary outcome is defined as a dichotomous outcome of whether the child has been perceived by Speech and Language Therapists (SLTs), following independent review of speech recordings, to have insufficient velopharyngeal function at age 5 years or not. Velopharyngeal insufficiency is measured by Velopharyngeal Composite Score (VPC) sum, which is a sum of scores, based on three components: hypernasality, non-oral errors, and velopharyngeal insufficiency (VPI) symptoms. Each component is classified and each classification mapped on to a score, see Table 1. The sum of the three scores, see Eq. 1, gives the VPC sum on the scale 0–6 [13]. Scores ≥ 4 on this scale will be considered insufficient.

**Table 1:** Calculating the VPC sum

Component	Classification	Score for component
Hypernasality	Within normal limits	0
	Mild resonance	1
	Moderate/severe resonance	2
Non-oral errors	0–2 errors	0
	3–5 errors	1
	≥ 6 errors	2
Velopharyngeal insufficiency symptoms	0–2 symptoms	0
	3–5 symptoms	1
	≥ 6 symptoms	2

*Equation 1: Using the three component scores to calculate VPC sum*

1 $$ \mathrm{VPC}\kern0.5em \mathrm{sum}=\kern0.5em \mathrm{Hypernasality}\kern0.5em \mathrm{score}+\mathrm{Active}\kern0.5em \mathrm{non}-\mathrm{oral}\kern0.5em \mathrm{errors}\kern0.5em \mathrm{score}+\mathrm{VPI}\kern0.5em \mathrm{symptoms}\kern0.5em \mathrm{score} $$

#### Secondary outcomes

Secondary outcome measures are defined in the following list. Outcomes 1 to 5 are a measure of speech development, which are classified by SLTs following independent review of speech recordings. Outcomes 6, 7, 8, and 10 are a measure of safety of the procedure, hearing level, middle ear function, and growth respectively and are measured at the relevant follow-up visits. Outcome 9 is a measure of dentofacial development measured independently on a profile photograph and maxillary arch impression taken during the 5-year follow-up visit.

1. *Velopharyngeal function at age 5 years:*
  a *Velopharyngeal composite score summary (VPC sum)*: a long ordinal outcome of individual score that contributes to the primary outcome, see Table 1. This score is measured on a scale of 0–6.
  b *Insufficient velopharyngeal function (VPC rate):* a dichotomous outcome of whether the child has “insufficient” VPC rate.
2. *Velopharyngeal function at age 3 years:*
  a *Insufficient velopharyngeal function (VPC rate):* a dichotomous outcome of whether the child has “insufficient” VPC rate.
  b *Velopharyngeal insufficiency symptoms:* a bounded continuous outcome, the proportion of times that a target consonant uttered has a velopharyngeal insufficiency symptom. Each child will attempt a minimum of 15 and a maximum of 30 predetermined target consonants (in words).
3. *Canonical babbling at age 12 months:*
  a *Canonical babbling present:* a dichotomous outcome of whether the child is “canonical” or “not canonical”.
  b *Canonical babbling ratio:* a bounded continuous outcome, the proportion of times that a syllable produced is “canonical”. Determined as the average proportion from the three SLTs undertaking independent review.
  c *Consonant inventory:* a continuous outcome of the number of unique consonants, identified by at least two of three SLTs undertaking independent review, uttered by a child.
4. *Articulation at age 3 years:* Each child is required to have attempted a minimum of 15 and a maximum of 30 predetermined target consonants (in words) for articulation assessment.
  a *Percent consonants correct (PCC):* a bounded continuous outcome, the proportion of times that a target consonant is uttered correct.
  b *Percent correct placement (PCP):* a bounded continuous outcome, the proportion of times that a target consonant has the correct place of articulation.
  c *Percent correct manner (PCM):* a bounded continuous outcome, the proportion of times that a target consonant has the correct manner of articulation.
  d *Non-oral consonant errors:* a bounded continuous outcome, the proportion of times that a target consonant is realised as a non-oral error.
  e *Oral consonant errors:* a bounded continuous outcome, the proportion of times that a target consonant is realised as an oral error.
5. *Articulation at age 5 years:* Each child is required to have attempted a minimum of 18 and a maximum of 36 predetermined target consonants (in words).
  a *PCC:* a bounded continuous outcome, the proportion of times that a target consonant is uttered correct.
  b *PCP:* a bounded continuous outcome, the proportion of times that a target consonant has the correct place of articulation.
  c *PCM:* a bounded continuous outcome, the proportion of times that a target consonant has the correct manner of articulation.
  d *Non-oral consonant errors:* a bounded continuous outcome, the proportion of times that a target consonant is realised as a non-oral error.
  e *Oral consonant errors:* a bounded continuous outcome, the proportion of times that a target consonant is realised as an oral error.
6. *Postoperative/long-term complications:*
  a *Dehiscence:* a dichotomous outcome of whether the child has a postoperative dehiscence, measured 48 h and 30 days postoperatively.
  b *Infection:* a dichotomous outcome of whether the child has a postoperative infection, measured 48 h and 30 days postoperatively.
  c *Evidence of fistula:* a dichotomous outcome of whether the child has a postoperative fistula, assessed as “Yes” or “Probably”, measured 30 days postoperatively and at 3 and 5 years of age*.*
7. *Hearing level:*
  a *At 12 months:*
    i *Abnormal Transient Otoacoustic Emission (TEOAE):* a dichotomous outcome of whether the child has abnormal TEOAE.
    j *Abnormal sound field audiometry:* a dichotomous outcome of whether the child has abnormal sound field audiometry. Abnormal sound field audiometry is indicated by a measurement of > 30 dB HL for at least one of four frequencies tested: 500 Hz, 1000 Hz, 2000 Hz, or 4000 Hz.
  b *At 3 and 5 years:*
    i *Abnormal pure tone audiometry in at least one ear:* a dichotomous outcome of whether the child has abnormal pure tone audiometry. If testing by pure tone audiometry is not possible, sound field audiometry can be used in its place. Abnormal audiometry in at least one ear is indicated by a measurement of > 20 dB HL using the pure tone method, > 25 dB HL for sound field, for at least one of four frequencies tested: 500 Hz, 1000 Hz, 2000 Hz, or 4000 Hz.
    j *Abnormal pure tone audiometry in both ears:* a dichotomous outcome defined in the same way as secondary outcome 7bi, for patients who have both ears tested and both tested ears indicate abnormal audiometry.
    k *Severity of better ear:* a short ordinal outcome of the severity of the better ear. If testing by pure tone audiometry is not possible, sound field audiometry can be used in its place. Each patient will be classified according to the average score in the better ear if pure tone, or both ears if sound field, across the four frequencies (500 Hz, 1000 Hz, 2000 Hz, or 4000 Hz) to the categories in Table 2 [14].
8. *Middle ear function:*
  a *Flat line tympanogram in at least one ear:* a dichotomous outcome of whether the child has flat line tympanogram, assessed at age 12 months, 3 years, and 5 years. Children with either ear measured as “Type B” will be classified as having flat line tympanogram in at least one ear.
  b *Flat line tympanogram in both ears:* a dichotomous outcome of whether the child has flat line tympanogram, assessed at age 12 months, 3 years, and 5 years. Children with both ears measured as “Type B” will be classified as having flat line tympanogram in both ears.
9. *Dentofacial development at age 5 years:*
  a *Soft tissue ANB angle:* a continuous outcome of the angle between soft tissue nasion (points A and B) measured using a profile photograph [15].
  b *Maxillary arch constriction score:* a bounded continuous outcome, measured using the Huddart/Bodenham scoring system, on a maxilliary and mandibular arch impression. A score can range from − 24 to 8 and is measured in whole numbers [16, 17]*.*
10. *Growth at 12 months:*
  a *Nude weight:* a continuous outcome, measured in grammes and recorded to the nearest whole number.
  b *Crown to heel length:* a continuous outcome, measured in centimetres and recorded to one decimal place.
  c *Occipitofrontal circumference:* a continuous outcome, measured in centimetres and recorded to one decimal place.

**Table 2:** Classifying severity in better ear

Average dB HL	Severity
≤ 20 dB HL	Normal
Between 21 and 40 dB HL	Mild
Between 41 and 70 dB HL	Moderate
Between 71 and 95 dB HL	Severe
> 95 dB HL	Profound

### Sample size

The sample size calculation was based on a test for proportions using a normal approximation: 292 participants per arm will allow a reduction in insufficient velopharyngeal function at 5 years from 40 to 29% to be detected with 80% power using a chi-squared test (2-sided significance test at 0.05 level). The estimate of 40% was obtained using data from a pilot trial of 50 5-year-old participants, conducted during the planning period for the grant application [18]. To allow an approximate attrition of 10%, 648 participants will be recruited. Restating the power for 300 participants per arm will allow the same difference to be detected with 81% power using a chi-squared test (2-sided significance test at 0.05 level). To consider the potential impact of variability around the value of 40%, 300 participants with valid data per group would provide 80% power to detect a reduction from 30 to 20% and 76% power to detect a reduction from 20 to 12%.

### Statistical analysis

#### General analysis principles

Three analysis populations will be considered: the intention-to-treat (ITT), the per-protocol (PP), and the safety population.

The principle of ITT, as far as practically possible, will be the main strategy of the analysis adopted for the primary outcome and all the secondary outcomes. These analyses will be conducted on all randomised participants, in the group to which they were allocated, and for whom the outcomes of interest have been observed/measured. No imputations are planned.

A per-protocol analysis, which will mirror the ITT population but exclude participants defined as having a major protocol deviation, will only be considered in the event of major protocol deviations in more than 10% of the ITT analysis population and apply to a secondary analysis of the primary outcome only. Table S1 provides a list of the protocol deviations.

The safety dataset will classify participants who have surgery before 9 months of gestational corrected age as received 6 months surgery, and surgery at 9 months of gestational corrected age or beyond as received surgery at 12 months.

A *p* value of 0.05 or less will be used to declare statistical significance for all analyses; *p* values will be reported to two significant figures. Rather than adjust for multiplicity, relevant results from other studies already reported in the literature will be taken into account when interpreting the study. Percentages will be presented to one decimal place, and continuous summary statistics will be given to a maximum of two decimal places.

All analyses will be performed using standard statistical software (SAS 9.4 or later). The finalised analysis datasets, programs, and outputs will be archived following Good Clinical Practice guidelines and standard operating procedures at the Liverpool Clinical Trials Centre.

#### Descriptive analyses

**Fig. 1:**
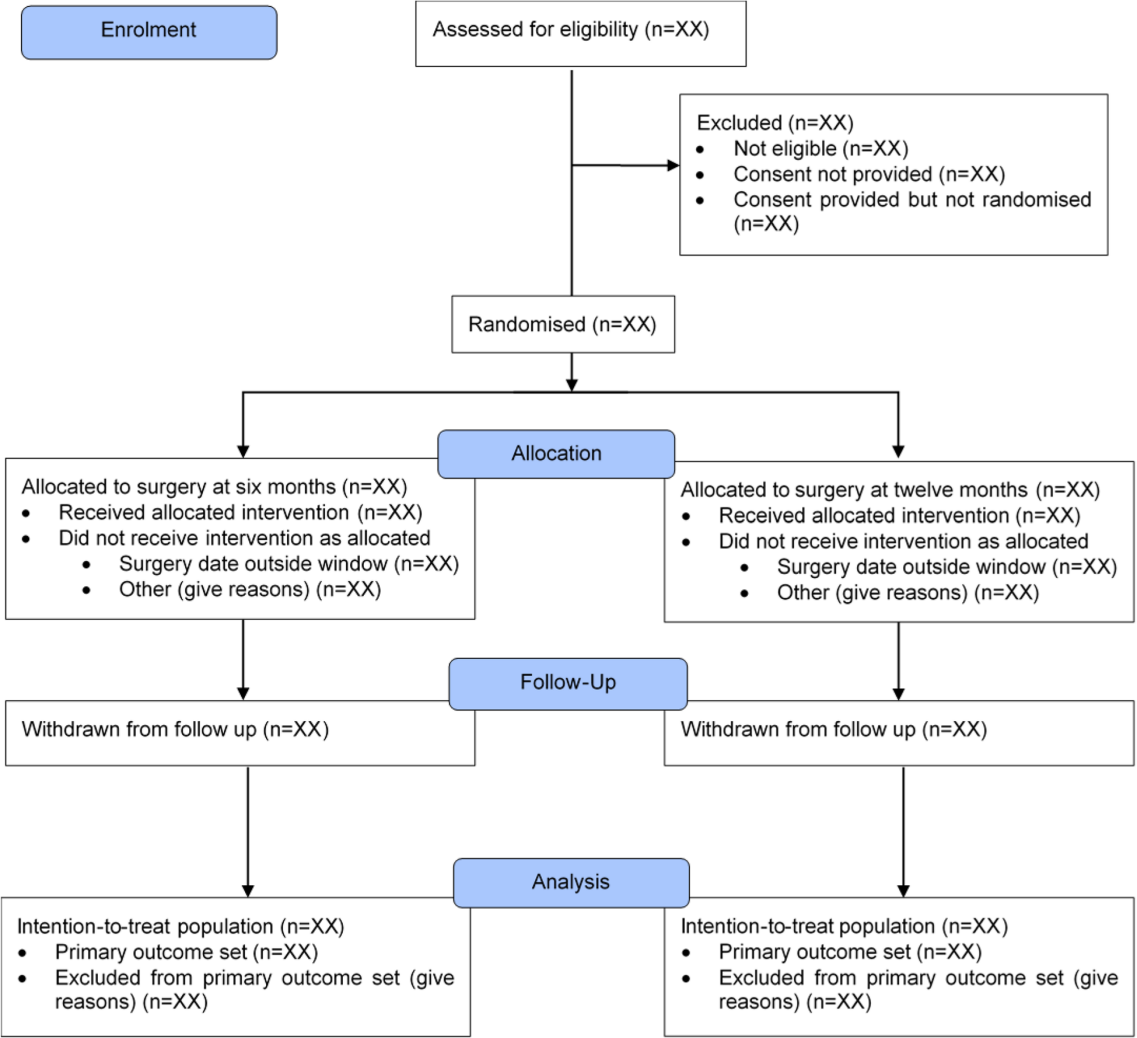
CONSORT flow diagram for participants in trial up to final assessment

The flow of participants through each stage of the trial, including the number of individuals screened, randomised, receiving treatment as allocated, and included in the primary analysis, will be summarised using a CONSORT flow chart [19] (Fig. 1).

The baseline comparability of the two randomised groups in terms of minimisation factors, demographic characteristics, and clinical genetics will be presented (Table 3).

**Table 3:** Baseline characteristics and clinical genetics

	6 months surgery	12 months surgery	Overall
Number of patients	XX	XX	XX
Baseline characteristics			
Gender			
*N*	XX	XX	XX
Male*; n (%)*	XX (XX.X)	XX (XX.X)	XX (XX.X)
Female*; n (%)*	XX (XX.X)	XX (XX.X)	XX (XX.X)
*Not known; n*	XX	XX	XX
Gestational age (weeks)			
*N*	XX	XX	XX
*Mean (SD)*	XX.X (XX.X)	XX.X (XX.X)	XX.X (XX.X)
*Median (IQR)*	XX.X (XX.X)	XX.X (XX.X)	XX.X (XX.X)
*(Min, max)*	(XX.X, XX.X)	(XX.X, XX.X)	(XX.X, XX.X)
*Not known; n*	XX	XX	XX
Size of cleft			
*N*	XX	XX	XX
*Soft palate only; n (%)*	XX (XX.X)	XX (XX.X)	XX (XX.X)
*Soft and hard palate; n (%)*	XX (XX.X)	XX (XX.X)	XX (XX.X)
*Not known; n*	XX	XX	XX
Clinical genetics			
Ethnicity			
*White; n (%)*	XX (XX.X)	XX (XX.X)	XX (XX.X)
*Black; n (%)*	XX (XX.X)	XX (XX.X)	XX (XX.X)
*Asian; n (%)*	XX (XX.X)	XX (XX.X)	XX (XX.X)
*Chinese; n (%)*	XX (XX.X)	XX (XX.X)	XX (XX.X)
*Mixed; n (%)*	XX (XX.X)	XX (XX.X)	XX (XX.X)
*Other; n (%)*	XX (XX.X)	XX (XX.X)	XX (XX.X)
*Not stated; n (%)*	XX (XX.X)	XX (XX.X)	XX (XX.X)
*Not known; n*	XX	XX	XX
Weight at examination (grammes)			
*N*	XX	XX	XX
*Mean (SD)*	XX.X (XX.X)	XX.X (XX.X)	XX.X (XX.X)
*Median (IQR)*	XX.X (XX.X)	XX.X (XX.X)	XX.X (XX.X)
*(Min, max)*	(XX.X, XX.X)	(XX.X, XX.X)	(XX.X, XX.X)
*Not known; n*	XX	XX	XX
Length at examination (cm)			
*N*	XX	XX	XX
*Mean (SD)*	XX.X (XX.X)	XX.X (XX.X)	XX.X (XX.X)
*Median (IQR)*	XX.X (XX.X)	XX.X (XX.X)	XX.X (XX.X)
*(Min, max)*	(XX.X, XX.X)	(XX.X, XX.X)	(XX.X, XX.X)
*Not known; n*	XX	XX	XX
Occipitofrontal circumference (cm)			
*N*	XX	XX	XX
*Mean (SD)*	XX.X (XX.X)	XX.X (XX.X)	XX.X (XX.X)
*Median (IQR)*	XX.X (XX.X)	XX.X (XX.X)	XX.X (XX.X)
*(Min, max)*	(XX.X, XX.X)	(XX.X, XX.X)	(XX.X, XX.X)
*Not known; n*	XX	XX	XX
Interpretation of DENVER-II			
*Normal; n (%)*	XX (XX.X)	XX (XX.X)	XX (XX.X)
*Suspect; n (%)*	XX (XX.X)	XX (XX.X)	XX (XX.X)
*Un-testable; n (%)*	XX (XX.X)	XX (XX.X)	XX (XX.X)
*Not known; n*	XX	XX	XX
Diagnosis			
*Known syndrome; n (%)*	XX (XX.X)	XX (XX.X)	XX (XX.X)
*Unknown syndrome; n (%)*	XX (XX.X)	XX (XX.X)	XX (XX.X)
*Severe developmental delay; n (%)*	XX (XX.X)	XX (XX.X)	XX (XX.X)
*Uncertain; n (%)*	XX (XX.X)	XX (XX.X)	XX (XX.X)
*Non-syndromic; n (%)*	XX (XX.X)	XX (XX.X)	XX (XX.X)
*Not known; n*	XX	XX	XX

The surgical comparability of the two randomised groups in terms of baseline surgery characteristics, intra-operative events, early complications during the hospital stay, observations monitored 48 h post-surgery, and postoperative medication will be presented (Table 4).

**Table 4:** Surgery characteristics and observations

	6 months surgery	12 months surgery	Overall
Number of patients	XX	XX	XX
Baseline surgery characteristics			
Grading of cleft at surgery			
*N*	XX	XX	XX
*Grade1; n (%)*	XX (XX.X)	XX (XX.X)	XX (XX.X)
*Grade 2; n (%)*	XX (XX.X)	XX (XX.X)	XX (XX.X)
*Grade 3; n (%)*	XX (XX.X)	XX (XX.X)	XX (XX.X)
*Grade 4; n (%)*	XX (XX.X)	XX (XX.X)	XX (XX.X)
*Not known; n*	XX	XX	XX
Shape of cleft			
*N*	XX	XX	XX
*“U” shaped; n (%)*	XX (XX.X)	XX (XX.X)	XX (XX.X)
*“V” shaped; n (%)*	XX (XX.X)	XX (XX.X)	XX (XX.X)
*Not known; n*	XX	XX	XX
Dimensions of cleft palate (mm)			
Soft tissue width at posterior hard plate			
*N*	XX	XX	XX
*Mean (SD)*	XX.X (XX.X)	XX.X (XX.X)	XX.X (XX.X)
*Median (IQR)*	XX.X (XX.X)	XX.X (XX.X)	XX.X (XX.X)
*(Min, max)*	(XX.X, XX.X)	(XX.X, XX.X)	(XX.X, XX.X)
*Not known; n*	XX	XX	XX
Bony width at posterior hard palate			
*N*	XX	XX	XX
*Mean (SD)*	XX.X (XX.X)	XX.X (XX.X)	XX.X (XX.X)
*Median (IQR)*	XX.X (XX.X)	XX.X (XX.X)	XX.X (XX.X)
*(Min, max)*	(XX.X, XX.X)	(XX.X, XX.X)	(XX.X, XX.X)
*Not known; n*	XX	XX	XX
Width at base of uvula			
*N*	XX	XX	XX
*Mean (SD)*	XX.X (XX.X)	XX.X (XX.X)	XX.X (XX.X)
*Median (IQR)*	XX.X (XX.X)	XX.X (XX.X)	XX.X (XX.X)
*(Min, max)*	(XX.X, XX.X)	(XX.X, XX.X)	(XX.X, XX.X)
*Not known; n*	XX	XX	XX
Length of soft palate (distal base of uvula-hard palate)			
*N*	XX	XX	XX
*Mean (SD)*	XX.X (XX.X)	XX.X (XX.X)	XX.X (XX.X)
*Median (IQR)*	XX.X (XX.X)	XX.X (XX.X)	XX.X (XX.X)
*(Min, max)*	(XX.X, XX.X)	(XX.X, XX.X)	(XX.X, XX.X)
*Not known; n*	XX	XX	XX
Intra-operative events			
Blood transfusion during surgery			
*N*	XX	XX	XX
*Yes; n (%)*	XX (XX.X)	XX (XX.X)	XX (XX.X)
*No; n (%)*	XX (XX.X)	XX (XX.X)	XX (XX.X)
*Not known; n*	XX	XX	XX
Anaesthetic complications			
*N*	XX	XX	XX
*Yes; n (%)*	XX (XX.X)	XX (XX.X)	XX (XX.X)
*No; n (%)*	XX (XX.X)	XX (XX.X)	XX (XX.X)
*Not known; n*	XX	XX	XX
Bleeding			
*N*	XX	XX	XX
*Yes; n (%)*	XX (XX.X)	XX (XX.X)	XX (XX.X)
*No; n (%)*	XX (XX.X)	XX (XX.X)	XX (XX.X)
*Not known; n*	XX	XX	XX
Early complications during the hospital stay			
Postoperative airway problems			
*N*	XX	XX	XX
*Yes; n (%)*	XX (XX.X)	XX (XX.X)	XX (XX.X)
*No; n (%)*	XX (XX.X)	XX (XX.X)	XX (XX.X)
*Not known; n*	XX	XX	XX
Postoperative blood loss			
*N*	XX	XX	XX
*Yes; n (%)*	XX (XX.X)	XX (XX.X)	XX (XX.X)
*No; n (%)*	XX (XX.X)	XX (XX.X)	XX (XX.X)
*Not known; n*	XX	XX	XX
Anti-coagulants given			
*N*	XX	XX	XX
*Yes; n (%)*	XX (XX.X)	XX (XX.X)	XX (XX.X)
*No; n (%)*	XX (XX.X)	XX (XX.X)	XX (XX.X)
*Not known; n*	XX	XX	XX
Dehiscence			
*N*	XX	XX	XX
*Yes; n (%)*	XX (XX.X)	XX (XX.X)	XX (XX.X)
*No; n (%)*	XX (XX.X)	XX (XX.X)	XX (XX.X)
*Not known; n*	XX	XX	XX
Readmission to operating room			
*N*	XX	XX	XX
*Yes; n (%)*	XX (XX.X)	XX (XX.X)	XX (XX.X)
*No; n (%)*	XX (XX.X)	XX (XX.X)	XX (XX.X)
*Not known; n*	XX	XX	XX
Observations monitored in first 48 h post-surgery			
Oxygen saturation levels			
*N*	XX	XX	XX
*Clinically significant abnormality; n (%)*	XX (XX.X)	XX (XX.X)	XX (XX.X)
*No clinically significant abnormality; n (%)*	XX (XX.X)	XX (XX.X)	XX (XX.X)
*No observation; n (%)*	XX (XX.X)	XX (XX.X)	XX (XX.X)
*Not known; n*	XX	XX	XX
Carbon dioxide and oxygen			
*N*	XX	XX	XX
*Clinically significant abnormality; n (%)*	XX (XX.X)	XX (XX.X)	XX (XX.X)
*No clinically significant abnormality; n (%)*	XX (XX.X)	XX (XX.X)	XX (XX.X)
*No observation; n (%)*	XX (XX.X)	XX (XX.X)	XX (XX.X)
*Not known; n*	XX	XX	XX
Arterial blood gases			
*N*	XX	XX	XX
*Clinically significant abnormality; n (%)*	XX (XX.X)	XX (XX.X)	XX (XX.X)
*No clinically significant abnormality; n (%)*	XX (XX.X)	XX (XX.X)	XX (XX.X)
*No observation; n (%)*	XX (XX.X)	XX (XX.X)	XX (XX.X)
*Not known; n*	XX	XX	XX
Heart rate			
*N*	XX	XX	XX
*Clinically significant abnormality and treatment required; n (%)*	XX (XX.X)	XX (XX.X)	XX (XX.X)
*Clinically significant abnormality and no treatment required; n (%)*	XX (XX.X)	XX (XX.X)	XX (XX.X)
*No clinically significant abnormality; n (%)*	XX (XX.X)	XX (XX.X)	XX (XX.X)
*No observation; n (%)*	XX (XX.X)	XX (XX.X)	XX (XX.X)
*Not known; n*	XX	XX	XX
Blood pressure			
*N*	XX	XX	XX
*Clinically significant abnormality and treatment required; n (%)*	XX (XX.X)	XX (XX.X)	XX (XX.X)
*Clinically significant abnormality and no treatment required; n (%)*	XX (XX.X)	XX (XX.X)	XX (XX.X)
*No clinically significant abnormality; n (%)*	XX (XX.X)	XX (XX.X)	XX (XX.X)
*No observation; n (%)*	XX (XX.X)	XX (XX.X)	XX (XX.X)
*Not known; n*	XX	XX	XX
Respiration			
*N*	XX	XX	XX
*Clinically significant abnormality; n (%)*	XX (XX.X)	XX (XX.X)	XX (XX.X)
*No clinically significant abnormality; n (%)*	XX (XX.X)	XX (XX.X)	XX (XX.X)
*No observation; n (%)*	XX (XX.X)	XX (XX.X)	XX (XX.X)
*Not known; n*	XX	XX	XX
Body temperature			
*N*	XX	XX	XX
*Clinically significant abnormality and treatment required; n (%)*	XX (XX.X)	XX (XX.X)	XX (XX.X)
*Clinically significant abnormality and no treatment required; n (%)*	XX (XX.X)	XX (XX.X)	XX (XX.X)
*No clinically significant abnormality; n (%)*	XX (XX.X)	XX (XX.X)	XX (XX.X)
*No observation; n (%)*	XX (XX.X)	XX (XX.X)	XX (XX.X)
*Not known; n*	XX	XX	XX
Postoperative medication			
Pain relief medication			
*N*	XX	XX	XX
*Yes; n (%)*	XX (XX.X)	XX (XX.X)	XX (XX.X)
*No; n (%)*	XX (XX.X)	XX (XX.X)	XX (XX.X)
*Not known; n*	XX	XX	XX
Anti-inflammatory medication			
*N*	XX	XX	XX
*Yes; n (%)*	XX (XX.X)	XX (XX.X)	XX (XX.X)
*No; n (%)*	XX (XX.X)	XX (XX.X)	XX (XX.X)
*Not known; n*	XX	XX	XX
Antibiotic medication (other than prophylactic antibiotics)			
*N*	XX	XX	XX
*Yes; n (%)*	XX (XX.X)	XX (XX.X)	XX (XX.X)
*No; n (%)*	XX (XX.X)	XX (XX.X)	XX (XX.X)
*Not known; n*	XX	XX	XX

Binary and categorical data will be summarised by frequencies and percentages. Continuous data will be presented by means and standard deviations (SDs), or medians and inter-quartile range (IQR) if data are skewed. Tests of statistical significance will not be undertaken for baseline characteristics; rather, the clinical importance of any imbalance will be noted. The amount missing in each case will be summarised.

#### Lost to follow-up, withdrawals, and missing data

The timing of withdrawal in relation to surgery and scheduled visits, level of withdrawal, who made the decision, and reason for withdrawal will be summarised both overall and for each randomised group. Frequencies will be presented along with percentages using the number of participants who withdrew as the denominator.

The number lost to follow-up both overall and within each randomised group will be reported, and the reasons where known will be documented. Any deaths and their causes will be reported separately.

Based on experience from the ScandCleft study, the structure of the centralised cleft palate care system and trial-specific systems in place will ensure the occurrence of missing data is likely to be low. Therefore, the potential impact of any missing data is likely to be low. For all assessments and outcomes, participants with insufficient data to make their assessments will be expressed as a frequency and a percentage with the denominator being those who were randomised, treated, and consented.

#### Adherence

Reasons for participants not receiving the randomised allocation will be summarised in a table. Adherence with follow-up time points (30 days, 12 months, 3 and 5 years) will be summarised at the visit level (at least one scheduled assessment visit completed per time point) and assessment level, which will specify adherence to specific assessments. When applicable, whether or not assessments were made within the expected window (Table 5) will be presented. Summaries will be presented both overall and for each randomised group.

**Table 5:** Follow-up visits: estimated visit dates and corresponding window limits

Time point	Estimated visit date, calculated from date of birth	Window limits of estimated visit date	
		Earliest	Latest
12 months	52 weeks^1^	*6 months arm:* − 4 weeks	+ 48 weeks (equivalent to 23 months of age)^2^
		*12 months arm:* − 2 weeks	
3 years	156 weeks	− 4 weeks	+ 48 weeks (equivalent to 3 years 11 months of age)
5 years	260 weeks	− 4 weeks	+ 48 weeks (equivalent to 5 years 11 months of age)

^1^Corrected for gestational age. Full term is defined as day 1 of the 40th week of pregnancy

^2^Speech assessments vary in their latest acceptable date:

• Six months surgery patients should have their speech assessed by + 26 weeks (equivalent to 18 months of age)

• Twelve months surgery should have their speech assessed before surgery

#### Analysis of primary outcome

The number of participants at age 5 years who have the primary outcome of insufficient velopharyngeal function (VPC sum ≥ 4) or not (VPC sum < 4) will be summarised overall, for each randomised group and for each region (defined according to the location of the recruiting site: Brazil, Scandinavia, UK) by frequencies and percentages. The numbers of insufficient velopharyngeal function or not between the randomised groups will be compared using a chi-squared test, with the relative risk [20] and 95% confidence interval (95% CI) also reported.

In the circumstance that the expected insufficient VPC sum or not for each randomised group contains less than five participants, thereby raising concerns over the appropriateness of a chi-squared test, then Fisher’s exact test will be used.

For inclusion in the primary analysis set, a participant must have an eligible 5-year speech recording for SLT assessment. An eligible speech recording requires the child to have attempted at least 18 of the 36 pre-specified target words for assessment of non-oral and VPI symptom and 5 of the 9 pre-specified words for hypernasality. Participants who have speech recordings that do not meet the inclusion criteria, have a recording that could not be assessed (e.g. insufficient sound), or did not complete a speech recording will be excluded from the analysis.

A sensitivity analysis will be performed to check the robustness of the results to the inclusion/exclusion set for speech recordings such that assessments made on:

- Non-oral and VPI symptom recordings with less than 18 target words attempted are included;
- +Hypernasality recordings with less than 5 target words attempted are included;
- Audio recordings, where video recording not possible, for non-oral and VPI symptom are excluded;
- Any recordings taken outside of the speech recording follow-up window (Table 5) are excluded.

A multilevel logistic regression model (for insufficient VPC sum) adjusting for operating surgeon, size of cleft at baseline (soft palate only vs. soft and hard palate), randomised group, and an intercept will be applied to check the robustness of the results to an unadjusted analysis approach [21–24].

An exploratory analysis will be undertaken where the primary endpoint of insufficient velopharyngeal function, defined as a score of 4–6 on VPC sum, also includes patients who have a secondary surgery due to velopharyngeal insufficiency. This group will be compared to patients who have a score of 0 to 3 on the VPC sum scale and have not received a secondary surgery due to velophyngeal insufficiency. This redefined binary endpoint will be compared using a chi-squared test, with the relative risk [20] and 95% CI also reported.

#### Analysis of secondary outcomes

Analysis approaches for each secondary outcome are dependent on the type of outcome; Table 6 provides a summary.

**Table 6:** Endpoint type for secondary outcomes

Outcome type	Outcome
Dichotomous	1b: Insufficient velopharyngeal function (VPC rate) at 5 years
	2a: Insufficient velopharyngeal function (VPC rate) at 3 years
	3a: Canonical babbling present at 12 months
	6a: Postoperative dehiscence
	6b: Postoperative infection
	6c: Evidence of fistula
	7ai: Abnormal Transient Otoacoustic Emission (TEOAE) at 12 months
	7aii: Abnormal sound field audiometry at 12 months
	7bi_1: Abnormal pure tone audiometry in at least one ear at 3 years
	7bi_2: Abnormal pure tone audiometry in at least one ear at 5 years
	7bii_1: Abnormal pure tone audiometry in both ears at 3 years
	7bii_2: Abnormal pure tone audiometry in both ears at 5 years
	8a_1: Flat line tympanogram in at least one ear at 12 months
	8a_2: Flat line tympanogram in at least one ear at 3 years
	8a_3: Flat line tympanogram in at least one ear at 5 years
	8b_1: Flat line tympanogram in both ears at 12 months
	8b_2: Flat line tympanogram in both ears at 3 years
	8b_3: Flat line tympanogram in both ears at 5 years
Short ordinal	7biii_1: Severity of better ear at 3 years
	7biii_2: Severity of better ear at 5 years
Long ordinal	1a: Velopharyngeal composite score summary (VPC sum) at 5 years
Bounded continuous	2b: Velopharyngeal insufficiency symptoms at 3 years
	3b: Canonical babbling ratio at 12 months
	4a: Percent consonants correct (PCC) at 3 years
	4b: Percent correct placement (PCP) at 3 years
	4c: Percent correct manner (PCM) at 3 years
	4d: Non-oral consonant errors at 3 years
	4e: Oral consonant errors at 3 years
	5a: Percent consonants correct (PCC) at 5 years
	5b: Percent correct placement (PCP) at 5 years
	5c: Percent correct manner (PCM) at 5 years
	5d: Non-oral consonant errors at 5 years
	5e: Oral consonant errors at 5 years
	9b: Maxillary arch constriction score at 5 years
Continuous	3c: Consonant inventory at 12 months
	9a: Soft tissue ANB angle at 5 years
	10a: Nude weight at 12 months
	10b: Crown to heel length at 12 months
	10c: Occipitofrontal circumference at 12 months

##### Dichotomous outcomes

For outcomes of this type, see Table 6, the number of participants categorised as having, or not having, the outcome of interest will be summarised overall, for each randomised group by frequencies and percentages. The numbers with the outcome between the randomised groups will be compared using a chi-squared test, with the relative risk [20] and 95% CI also reported.

In the circumstance that the expected number of participants with and without the outcome for each randomised group contains less than five participants, thereby raising concerns over the appropriateness of a chi-squared test, then Fisher’s exact test will be used.

No sensitivity analysis will be performed.

##### Short ordinal outcomes

For outcomes of this type, see Table 6, the number of participants who are categorised into each classification of interest will be summarised overall, for each randomised group by frequencies and percentages. The numbers with the outcome between the randomised groups will be compared using a chi-squared test for trend.

In the circumstance that the expected number of participants in each classification for each randomised group contains less than five participants, thereby raising concerns over the appropriateness of a chi-squared test for trend, an alternative appropriate analysis approach will be used, e.g. combining like groups or applying a proportional odds model.

No sensitivity analysis will be performed.

##### Long ordinal, bounded continuous, and continuous outcomes

Outcomes of this type, see Table 6, will be summarised overall and for each randomised group by means and SDs, or medians and IQRs if data are skewed. Minimum and maximum values will also be presented.

Means will be compared between the two randomised groups using a *t* test or by using a non-parametric equivalent. Testing for normality of data distributions will be based using a QQ plot by randomised group. Ninety-five percent confidence intervals will be presented around the effect measure.

No sensitivity analysis will be performed.

#### Inter- and intra-rater reliability

Each of the outcomes assessed by independent assessors, i.e. not at routine visit (outcomes 1–5 and 9), will each be reviewed by a minimum of one assessor and a maximum of three. Intra and inter assessments will be undertaken for a proportion of all outcomes to ensure reliability of the outcome measures. The number of assessors and proportion of inter- and intra-rater assessments is determined a priori and decided on a per-outcome basis, led by members of the Trial Management Group (see http://www.tops-trial.org.uk/) who are specialists in the specific outcome field.

Agreement analysis will be exploratory and report agreement as frequencies and percentages or using Bland-Altman agreement analysis as appropriate for the outcome type [25].

#### Additional analyses

To support interpretation of the main trial outcomes, descriptive statistics will be summarised to report the results of the following: (i) the DENVER-II test at 3 years, (ii) additional speech therapy received outside of routine trial visits, (iii) reasons and the nature of any secondary surgeries received during the trial, and (iv) nasometry at 5 years. Binary and categorical data will be summarised by frequencies and percentages. Continuous data will be presented by means and SDs, or medians and IQR if data are skewed.

Tests of statistical significance will not be undertaken for (i), (ii), and (iii); rather, the clinical importance of any imbalance will be noted. The amount missing in each case will be summarised. (iv) Nasometry, at age 5 years, will be compared between the two randomised groups using a *t* test or by using a non-parametric equivalent. Testing for normality of data distributions will be based using a QQ plot by randomised group. Ninety-five percent confidence intervals will be presented around the effect measure.

#### Safety evaluations

Serious adverse events and unanticipated problems will be presented using descriptive statistics. Line listings of events will also be presented to provide further detail. Patients will be reported according to the safety dataset, with the number of events and patients in each safety group summarised. Tests of statistical significance will not be undertaken; rather, the clinical importance of any imbalance will be noted.

## Discussion

The TOPS trial will provide evidence to support whether surgery for cleft palate at age 6 months when compared to surgery at age 12 months improves velopharyngeal function at age 5 years. In addition, evidence regarding a wide range of pre-defined clinical secondary outcomes will be explored. This paper provides details of the planned statistical analyses of the trial. Publishing these plans prior to trial results will improve the scientific validity of the TOPS trial and reduce the risk of outcome reporting bias and data-driven results [26].

## Trial status

The trial completed recruitment on 21 July 2015. In total, 558 patients from 22 centres were recruited and the last patient is due to attend their last visit on 30 July 2020. The analysis of outcomes will be conducted thereafter.

## Supplementary Information

**Additional file 1: Table S1.** Definition of protocol deviations.

## Data Availability

Not applicable.

## Abbreviations

95% CI: 95% confidence interval
IQR: Inter-quartile range
ITT: Intention-to-treat
PCC: Percent consonants correct
PCM: Percent correct manner
PCP: Percent consonants placement
PP: Per-protocol
SD: Standard deviation
SLT: Speech and Language Therapist
TOPS: Timing Of Primary Surgery for cleft palate
VPC: Velopharyngeal Composite Score
VPC rate: Insufficient velopharyngeal function
VPC sum: Velopharyngeal Composite Score summary
