# Supplementary material for: A randomised controlled trial comparing palate surgery at 6 months versus 12 months of age (the TOPS trial): a statistical analysis plan
Source: Trials. 2021 Jan 4;22:5. doi: 10.1186/s13063-020-04886-y (PMC7780678; doi:10.1186/s13063-020-04886-y)
Supplement: Supplementary file 1 — Additional file 1: Table S1. Definition of protocol deviations. [file 13063_2020_4886_MOESM1_ESM.docx]

**SUPPLEMENTARY MATERIAL**

Table S1: Definition of protocol deviations

| Protocol specification | Potential deviation(s) | Impact | Justification |
| --- | --- | --- | --- |
| Inclusion criteria | | | |
| Written informed proxy consent | Consent not obtained | **Major** | Violation of participant rights |
| One parent/carer a native language speaker | Neither parent or carer a native language speaker | **Major** | Statistical impact  Primary outcome (insufficient velopharyngeal function) may be biased to poorer outcome in these patients.  Primary analysis will be biased if violation is imbalanced between treatment groups.  Safety impact  None |
| Exclusion criteria |  |  |  |
| Infants with syndromic cleft palate or severe developmental delay | Identification post randomisation | **Major** | Statistical impact  Primary outcome (insufficient velopharyngeal function) may be biased to poorer outcome in these patients or may not be evaluable resulting in missing data.  Primary analysis will be biased if occurrence of violation is imbalanced between treatment groups or a reduction in power caused by missing data.  Safety impact  None |
| Congenital sensorineural hearing loss or structural middle ear anomalies | Identification post randomisation | **Major** | Statistical impact  Primary outcome (insufficient velopharyngeal function) may be biased to poorer outcome in these patients.  Primary analysis will be biased if occurrence of violation is imbalanced between treatment groups.  Safety impact  None |
| One stage closure with the Sommerlad technique would be inappropriate | One stage closure with the Sommerlad technique not used at study surgery | **Major** | Statistical impact  This will introduce greater variation in definition of the study treatment.  This may bias the primary outcome.  Unknown whether it will increase variation in the primary outcome.  Safety impact  No greater than standard medical care where the most appropriate surgical technique will be used. |
| Submucous cleft palate | Submucous cleft palate is a different type of cleft palate condition than the study targeted isolated overt cleft palate condition | **Major** | Statistical impact  Interpretations of results are intended only for isolated overt cleft palate medical condition. The inclusion of other types of cleft conditions in study summaries will add uncertainty in interpretation for the intended isolated overt cleft palate population. In addition, the presence of other cleft types may increase heterogeneity in the primary outcomes and therefore potential to decrease statistical power.  Safety impact  None |
| Treatment: timing of surgery | |  |  |
| Surgery at six months or twelve months should be within visit windows | Surgery outside visit window | **Major** | Statistical impact  Patients would no longer be receiving the study intervention defined in protocol.  Safety impact  Major impact if a child was operated on earlier than assigned timing of surgery. In particular, surgery earlier than 6 months is expected to carry the highest risk of peri-operative complications.  Minor or no impact if surgery was conducted later than assigned timing of surgery. |
| Withdrawal before surgery due to parent/guardian decision | (a) Refusal to keep to assigned timing of surgery  (b) Withdrawal with no follow up | **Major** | Statistical impact  (a) Bias to treatment comparison as choice of treatment is non-randomised  (b) Missing data if not followed up  Safety impact  None |
| Study assessments |  |  |  |
| Scheduled assessment visit at 30 days post-surgery: late complications | Missing or follow up assessment deviates from 30 day post-surgery date | **Minor** | Statistical impact  No risk of bias to primary outcome (insufficient velopharyngeal function at age three and five years)  Safety impact  No impact. No visit window is specified in the protocol, but the number of day’s deviation from 30 days will be described. |
| Scheduled assessment visit at twelve months | Missing or visit occurs outside allowed window either side of visit date, or for twelve month patients occurs after surgery | **Minor** | Statistical impact  No risk of bias to primary outcome (insufficient velopharyngeal function at age three and five years).  However, there will be impact to secondary speech outcomes if speech visits are conducted in the twelve month group after the surgery has taken place the child is likely to be uncooperative or not perform as well as the surgery will be healing.  Safety impact  Minor for six month patients as surgery already performed.  Minor for twelve month patients as timing of scheduled assessments are scheduled around the surgery date and not vice versa, in order to keep to the study timing of surgery. |
| Scheduled assessment visit at three years and five years | Missing or visit occurs outside allowed window either side of visit date. | **Major** | Statistical impact  Data from both three and five years required for primary outcome.  Loss of power and potential for bias.  Safety impact  Minor impact as surgery already performed. |
